# Induced androgenetic development in rainbow trout and transcriptome analysis of irradiated eggs

**DOI:** 10.1038/s41598-019-44568-7

**Published:** 2019-05-30

**Authors:** Konrad Ocalewicz, Artur Gurgul, Klaudia Pawlina-Tyszko, Tomasz Szmatoła, Igor Jasielczuk, Monika Bugno-Poniewierska, Stefan Dobosz

**Affiliations:** 1 Department of Marine Biology and Ecology, Institute of Oceanography, Faculty of Oceanography and Geography, University of Gdansk, Av. M. Piłsudskiego 46, 81-378 Gdynia, Poland; 2 Department of Animal Molecular Biology, National Research Institute of Animal Production, 32-083 Balice, Krakowska 1, Poland; 3 University Centre of Veterinary Medicine UAK, University of Agriculture in Krakow, Al. Mickiewicza 24/28, 30-059 Krakow, Poland; 4 Institute of Veterinary Sciences, University of Agriculture in Krakow, Mickiewicza 24/28 Av, 30-059 Krakow, Poland; 5 Department of Salmonid Research, Inland Fisheries Institute in Olsztyn, Rutki, 83-330 Żukowo Poland

**Keywords:** Genetic engineering, Genetic engineering, Embryonic induction, Embryonic induction, Embryology

## Abstract

Ionizing radiation is administered to damage nuclear genome in fish eggs during induced androgenesis. In this study, we examined whether 350 Gy of X-ray applied to damage chromosomes in the rainbow trout eggs affects maternal RNA. Shortly after irradiation, we did not find any symptoms of RNA degradation in the treated eggs. Significant (p < 0.01) differences between non-irradiated and irradiated eggs concerned only a few transcripts including increased expression of immediate early response 2 (*IER2*) and early growth response 1 (*EGR1*) genes observed in the irradiated eggs. Both genes belong to the group of “immediate early genes” that respond quickly to the diverse extracellular stimuli. Elevated expression of these genes was accompanied by decreased level of ssa-miR-10b-5p and ssa-miR-21b-5p (p < 0.05), for which *IER2* and *EGR1* are target genes. The level of RNA in the fertilized irradiated eggs was highly significantly lower than in the non-irradiated eggs (p < 0.001) and in the unfertilized irradiated eggs (p < 0.0001). However, transcriptome profiles of fertilized non-irradiated eggs and fertilized irradiated eggs did not differ significantly. Thus, we assume that reduced abundance of mRNA in the fertilized irradiated eggs was associated with post-translational degradation and clearance of the maternal transcripts rather than from the irradiation of eggs.

## Introduction

Ionizing radiation has been used in the developmental biology and aquaculture to inactivate nuclear DNA in fish gametes to produce individuals with an exclusive paternal (androgenesis) or maternal (gynogenesis) nuclear genome^1–3^. Irradiated and fertilized eggs develop into haploid (1n) androgenetic embryos whereas, activation of untreated eggs with irradiated spermatozoa leads to the development of gynogenetic haploids. Exposure of androgenetic and gynogenetic haploid eggs to high hydrostatic pressure (HHP) or thermal shock during prophase of the first mitotic division results in the inhibition of cell cleavage, duplication of haploid sets of chromosomes and production of diploid (2n) androgenetic and gynogenetic specimens known as Doubled Haploids (DHs)^3^. Haploid and Doubled Haploid individuals are useful in recessive genetic screening. Haploid fish embryos are the source of haploid embryonic stem cells^2^ while DH fish have been used in the breeding programs, generation of monosex fish stocks, production of cloned fish and procedures of gene mapping and genome sequencing^3^.

Among salmonid fishes, the androgenetic haploid or doubled haploid development has been induced in a few species, including amago salmon (*Oncorhynchus masou ishikawae*)^4^, brown trout (*Salmo trutta*)^5^, brook trout (*Salvelinus fontinalis*)^6,7^, Arctic charr (*Salvelinus alpinus*)^7^ and hybrids of these two *Salvelinus* species^8^. However, the most advanced research concerning androgenesis has been performed on the rainbow trout (*Oncorhynhcus mykiss*). Application of spermatozoa from androgenetic males for another round of androgenesis resulted in the establishment of rainbow trout clonal lines^9^. In the rainbow trout, successful androgenesis has been induced using cryopreserved sperm^10^, which makes this process a biotechnological tool enabling the reconstruction of nuclear genome information from cryobanked spermatozoa. Moreover, rainbow trout androgenetic haploid embryos were used in studies concerning the role of sex chromosomes during an early development^11^.

Unfortunately, the high mortality rate observed among androgenetic specimens is still a limiting factor for large-scale applications of androgenesis. Usually, less than 5% of androgenetic DHs hatch and only few of them survive till maturation^3,5–9^. The reduced survival of androgenotes results primarily from the expression of recessive alleles. However, irradiation of eggs may also impair their developmental competences. The early development of fish embryos is governed by the maternal RNA deposited in the egg cytoplasm during oocyte maturation. Zygotic Genome Activation (ZGA) is initiated only during the mid-blastula transition (MBT) that occurs around the 10^th^ cell cleavage^12^. It cannot be ruled out that ionizing radiation damages not only maternal chromosomes but also maternal RNAs, which are indispensable for the first cell cleavages in the fish embryos. On the other hand, X-ray applied to damage the nuclear DNA in fish eggs may also activate expression of genes involved in a DNA damage response (DDR), which includes a set of DNA repair mechanisms, damage tolerance processes and cell-cycle checkpoint pathways^13^. Recent studies have evidenced that haploid brown trout (*S. trutta*) embryos developing in untreated eggs exhibited a significantly lower mortality rate compared to haploids developing in irradiated eggs^14^. These observations suggest that exposure to ionizing radiation impairs the developmental potential of irradiated fish eggs due to changes in the maternal transcriptome. To verify this assumption, the quality and quantity of RNA extracted from rainbow trout eggs and subjected to ionizing radiation for the androgenetic purpose were studied using standard molecular techniques and the RNAseq approach.

## Results

**Figure 1:**
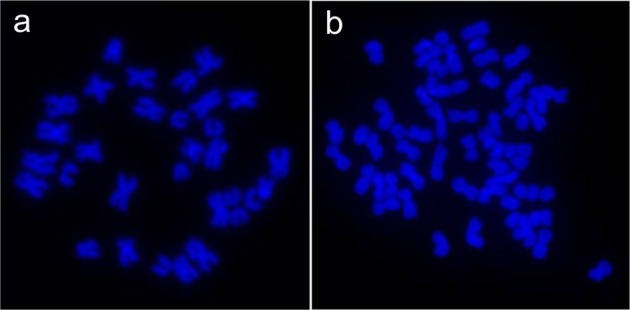
Metaphase spreads of haploid (1n = 31) (androgenetic) (**A**) and diploid (2n = 62) rainbow trout embryos (**B**).

Cytogenetic examination confirmed the haploid status of androgenetic rainbow trout individuals. Embryos that were developing in the irradiated eggs had 30 and 31 chromosomes (1n), while the number of chromosomes in specimens developing in non-irradiated eggs fertilized by normal spermatozoa varied from 60 to 62 (2n) (Fig. 1).

The survival of the haploid androgenetic rainbow trout at the eyed stage (embryogenesis) varied from 49.2 to 59.6%. The mortality of androgenotes increased dramatically and survival rates at hatching ranged between 6.25 and 13.1%. None of the androgenetic haploid larvae survived till the swim-up stage. Survival rates of the diploid individuals from the control groups exceeded 94% during the embryonic development and 91% at the swim-up stage.

### The effect of irradiation on the quantity and quality of RNA

**Figure 2:**
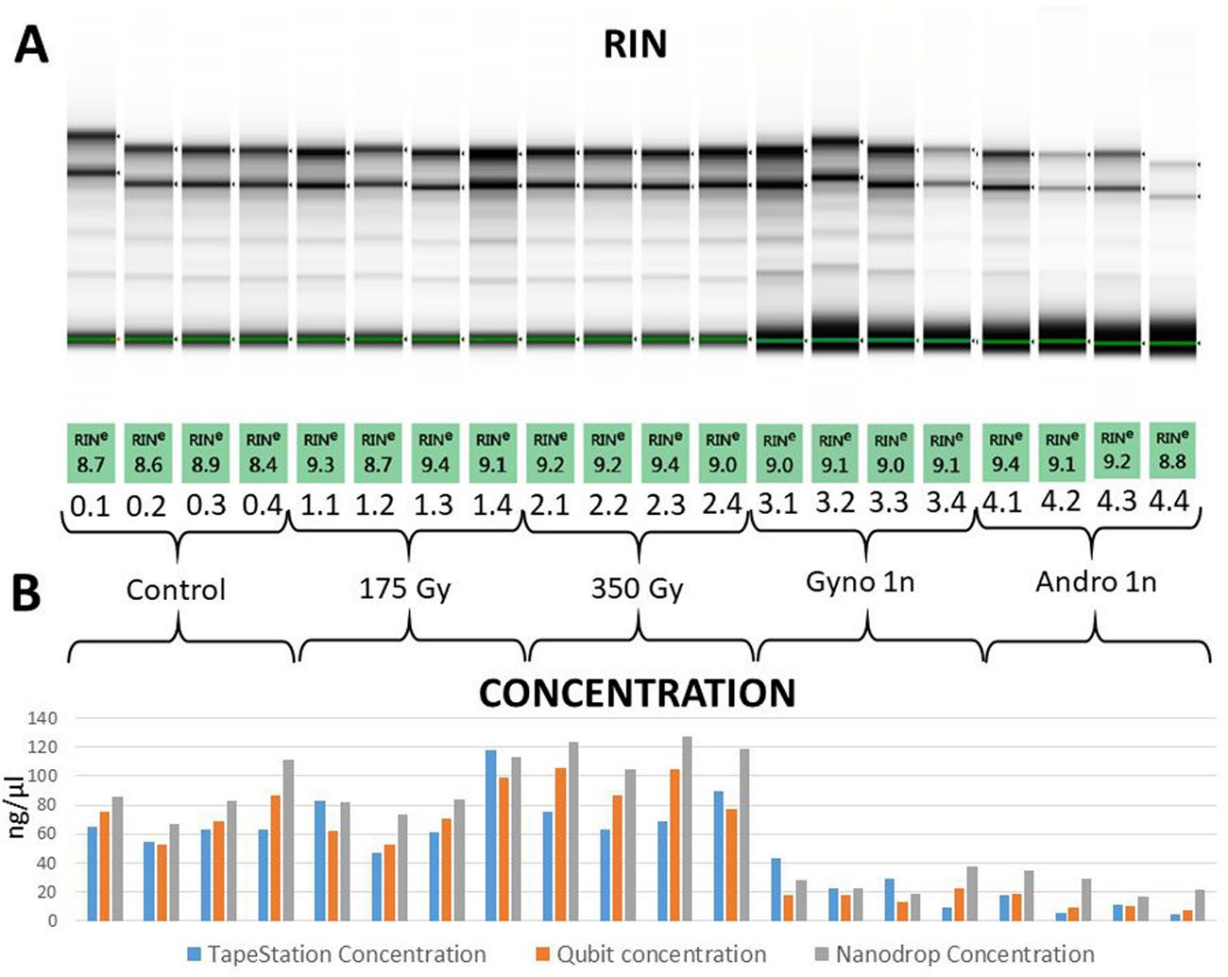
RNA integrity (quality) (**A**) and concentrations (**B**) obtained from non-irradiated eggs (control) and irradiated eggs (175 Gy, 350 Gy) before activation and non-irradiated and irradiated eggs after activation (Gyno 1n and Andro 1n, respectively). Electronically simulated gel images come from a few different runs in the TapeStation2200 System. However, after standardization and comparison between different experiments (https://www.agilent.com/cs/library/applications/5989–1165EN.pdf), the RIN values are presented in a common image (see material and methods for details).

The effect of irradiation on the quantity and quality of the purified total RNA was assessed by comparing the RNA concentrations determined by three different methods and RNA integrity measured with an RIN integer. In addition to standard spectrophotometric quantification, we used two different fluorometric methods to avoid quantification bias caused by the absorbance of contaminants of soluble isolates. No significant differences in RNA concentrations, obtained from a similar amount of material, were found between non-irradiated eggs and eggs treated with both 175 Gy and 350 Gy, when using two most reliable fluorometric methods (Fig. 2; Table 1). However, we observed some tendency toward the higher amount of RNA present in eggs irradiated with 350 Gy (especially when compared to non-irradiated eggs). The RNA quality was found to be significantly higher (p < 0.01) in eggs treated with 350 Gy (average RIN = 9.2 ± 0.16) compared to non-irradiated eggs (RIN = 8.6 ± 0.21; Table 1).

**Table 1:** RNA integrity (quality) and concentrations obtained from non-irradiated and irradiated eggs before activation and non-irradiated and irradiated eggs after activation (Gyno 1n and Andro 1n, respectively).

Samples		TapeStation RIN			T-test p-value*				TapeStation Concentration			Qubit concentration			T-test p-value*				Nanodrop Concentration		
Group	Sample	RIN	Mean	SD	175 Gy	350 Gy	gyno 1n	andro 1n	ng/µl	Mean	SD	ng/µl	Mean	SD	175 Gy	350 Gy	gyno 1n	andro 1n	ng/µl	Mean	SD
non-irradiated eggs	0.1	8.7	8.6	0.21	0.0437	0.0060	0.0100	0.0266	64.8	61.2	4.8	74.9	70.7	14.4	0.9753	0.0652	0.0004	0.0002	85.7	86.8	18.3
	0.2	8.6							54.1			52.3							67.1		
	0.3	8.9							62.6			68.7							83.2		
	0.4	8.4							63.3			86.9							111.3		
eggs irradiated with 175 Gy	1.1	9.3	9.1	0.31	x	0.6832	0.6507	1	82.7	77.3	30.8	62.2	71.1	20.1	x	0.1188	0.0020	0.0012	81.6	88.2	17.4
	1.2	8.7							47.4			52.6							73.7		
	1.3	9.4							61			70.3							83.9		
	1.4	9.1							118			99.3							113.5		
eggs irradiated with 350 Gy	2.1	9.2	9.2	0.16		x	0.1340	0.6333	75.5	74.1	11.5	106	93.6	14.3		x	5E-05	3E-05	124.1	118.8	9.7
	2.2	9.2							62.6			86.4							105.1		
	2.3	9.4							68.8			105							127		
	2.4	9							89.5			76.9							118.9		
Gyno 1n	3.1	9	9.1	0.06			x	0.58	43.4	26.0	14.4	17.7	17.7	3.9			x	0.08	28.2	26.6	8.5
	3.2	9.1							22.1			17.5							22.5		
	3.3	9							29.4			13							18.1		
	3.4	9.1							9			22.5							37.7		
Andro 1n	4.1	9.4	9.1	0.25				x	18	9.5	6.3	18.2	11.3	4.7				x	35.1	25.8	8
	4.2	9.1							5.5			9.1							29.3		
	4.3	9.2							10.6			10.1							17		
	4.4	8.8							4			7.6							21.8		

^*^T-test p-value was shown only for RIN and Qubit measurements.

### RNA-Seq and RNA sequencing statistics

Sequencing of mRNA libraries and filtering of reads allowed us to produce 16.3 to 42.9 million reads per sample, of which approximately 63.3% were mapped to the rainbow trout transcriptome. The number of detected expressed transcripts (normalized read count >1) averaged per group ranged from 30,136 to 28,150 in the non-irradiated eggs and in the inseminated irradiated eggs (Andro 1n), respectively (Supplementary Table 1). The sequencing of miRNA libraries allowed us to produce on average 4.89 million raw reads per sample and on average 1.85 million reads after subsequent filtering (Supplementary Table 2). The mRNA and miRNA sequencing data were deposited in the SRA database (Sequence Read Archive) under the accession number SRP156714.

### Assessment of changes in the relative transcript levels after irradiation of eggs

Irradiation of eggs with 175 Gy resulted in a differential expression of a very few genes. On the genome-wide level (p < 0.01), highly statistically significant differences between irradiated and non-irradiated eggs were observed and concerned four transcripts that were found to be upregulated in eggs treated with X-rays. These transcripts belonged to two genes that were identified as: immediate early response 2 (*IER2*) gene and early growth response 1 (*EGR1*) gene *Salmo salar* orthologs (Supplementary File 1). The irradiation of eggs with 350 Gy resulted in the upregulation of the same transcripts and downregulation of transcript of the Nicotinamide riboside kinase 2 gene (*NMRK2*) (Supplementary File 1). On the pointwise level (p < 0.05), both radiation doses affected transcript levels of 89 (175 Gy) and 754 (350 Gy) genes, including e.g. centrosomal protein of 85 kDa-like (*CEP85L*) gene, periphilin-1 (*PPHLN1*) gene and butyrate response factor 1 (*ZFP36L1*) gene (Supplementary File 1). No genome-wide significant differences in the transcript levels between eggs irradiated with 175 Gy and 350 Gy were found. However, on the pointwise level, transcripts of 107 genes were affected, with the most pronounced changes observed in eggs irradiated with 350 Gy and included downregulation of immunoglobulin mu heavy chain and kappa chain genes (Supplementary File 1).

### Changes in the miRNA expression profile in irradiated eggs

On the genome-wide level, irradiation of rainbow trout eggs with 175 Gy did not result in significant alteration of the microRNAs expression when compared to the control eggs. However, on the pointwise level, 25 known microRNAs and 6 potentially novel microRNAs were found to be differentially expressed in irradiated and non-irradiated eggs. The most statistically significant changes concerned the downregulation of ssa-miR-30b-5p and the upregulation of ssa-miR-22a-3p in the irradiated eggs. Moreover, differentially expressed miRNAs included ssa-miR-10b-5p and ssa-miR-21b-5p, both targeting *IER2* and *EGR1* mRNAs whose altered expressions were also observed in this study. On the pointwise level, miRNAs from the let-7 family were also deregulated after irradiation of eggs (Supplementary File 2).

The irradiation of eggs with a dose of 350 Gy resulted in differential expression of eight known and 14 potentially new miRNAs. However, these changes were significant only at the pointwise level. Eggs from both irradiation variants exhibited downregulation of isomiRs of ssa-miR-148a-3p. Among deregulated miRNAs, ssa-miR-146a-5p, ssa-miR-202-3p and ssa-miR-22a-3p isomiRs were overexpressed, whereas ssa-miR-206-3p, ssa-miR148a-3p, ssa-miR-199a-3p and ssa-miR-146d-5p isomiRs were under-expressed (Table 2, Supplementary File 2). Moreover, different isomiR variants of ssa-let-7b-5p exhibited up- or downregulation (Table 2, Supplementary File 2). When it comes to potentially new miRNAs, only three of the 14 miRNAs were differentially expressed in the 175 Gy irradiation group, while the remaining 11 miRNAs were exclusively deregulated in the 350 Gy group (Supplementary File 2).

**Table 2:** Most differentially expressed known miRNAs in the rainbow trout eggs exposed to ionizing radiation before activation (175Gy, 350 Gy) and after androgenetic activation (Andro 1n).

Exp. variant	miR name	miRNA sequence	Log2 (fold_change)	induced	repressed
175 Gy	ssa-miR-30b-5p	UGUAAACAUCCCCGACUGGAAGCG	−6.86		+
	ssa-miR-22a-3p	AAGCUGCCAGCCGAAGAACUGC	5.83	+	
	ssa-miR-22a-3p	AAGCUGCCAGCUGAAGAAU	5.7	+	
	ssa-miR-21b-5p	UAGCUUAUCAGACUGAUGUUG	−6.35		+
	ssa-miR-10b-5p	UACCCUGUAGAACCGAAUUUGUGA	−6.9		+
	ssa-let-7b-5p	UGAGGUAGUAGGUUGUGUGA	5.17	+	
	ssa-miR-10b-5p	UACCCUGUAGAACCGAAUU	−5.72		+
	ssa-miR-146a-5p	UGAGAACUGAAUUCCGUAGAUGG	−5.63		+
	ssa-miR-101a-3p	UACAGUACUGUGAUAACUGAAU	−6.86		+
	ssa-miR-206–3p	UGGAAUGUAAGGAAGUGUG	−6.86		+
350 Gy	ssa-miR-146a-5p	UGAGAACUGAAUUCCAUAGAUU	5.28	+	
	ssa-miR-206-3p	UGGAAUGUAAGGAAGUGUGUGAU	−6.33		+
	ssa-miR-202-3p	UUCCUAUGCAUAUACCCCUUC	4.96	+	
	ssa-miR-148a-3p	UCAGUGCAUCACAGAACUUUGUU	−6.16		+
	ssa-let-7b-5p	UGAGGUAGUAGGUUGUGUGA	4.85	+	
	ssa-let-7b-5p	UGAGGUAGUAGGUUGUGUGGUUC	−6.07		+
	ssa-miR-199a-3p	ACAGUAGUCUGCACAUUGGUG	−5.02		+
	ssa-miR-146d-5p	UGAGAACUGAAUUCCAUGGGUU	−5.98		+
	ssa-miR-22a-3p	AAGCUGCCAGCCGAAGAACUGC	4.79	+	
Andro 1n	ssa-let-7f-5p	UGAGGUAGUAGAUUGUAUAGUU	3.79	+	
	ssa-miR-133a-3p	UUUGGUCCCCUUCAACCAGCUGU	3.87	+	
	ssa-miR-133a-3p	UUGGUCCCCUUCAACCAGCUGU	3.64	+	
	ssa-miR-206-3p	UGGAAUGUAAGGAAGUGUGUGA	3.68	+	
	ssa-miR-133a-3p	UUGGUCCCCUUCAACCAGCUGUU	4.17	+	
	ssa-let-7g-5p	UGAGGUAGUAGUUUGUACAGUU	3.46	+	
	ssa-miR-133b-3p	UUUGGUCCCCUUCAACCAGCUGC	3.99	+	
	ssa-let-7g-5p	UGAGGUAGUAGUUUGUACAGU	3.73	+	
	ssa-let-7g-5p	UGAGGUAGUAGUUUGUACAGUC	3.97	+	
	Ssa-let-7f-5p	UGAGGUAGUAGAUUGUAUAGUC	4.00	+	
	ssa-miR-148a-3p	UCAGUGCACUACAGAACUUUGU	3.08	+	

At the genome-wide level, differences in the expression of miRNAs between eggs treated with 175 Gy and 350 Gy were insignificant. At the pointwise level, however, the expression of seven known and 13 potentially novel miRNAs were altered in eggs from different irradiation variants (Supplementary File 2). This comparison also revealed that only the lower dose of radiation changed the expression levels of ssa-miR-10b-5p and ssa-miR-21a-5p compared to non-irradiated eggs (log2 Fold Change −6.89 and −6.03, respectively).

### Transcriptome alterations in irradiated and non-irradiated eggs before and after fertilization

The quality and quantity of RNA and transcriptome alterations were also evaluated in non-irradiated and irradiated eggs before and after insemination (fertilization). For all RNA quantification methods, the level of RNA in activated irradiated (Andro 1n) and activated non-irradiated (Gyno 1n) eggs was highly significantly lower (p < 0.0001) when compared to irradiated eggs before insemination and to non-irradiated eggs (p < 0.001). No clear differences were found in mRNA integrity after the fertilization of irradiated eggs (RIN ~9.2) (Table 1). Average RINs of 8.65 and 9.12 were detected in the non-irradiated eggs and fertilized irradiated eggs, respectively (p < 0.05; Fig. 2; Table 1).

**Figure 3:**
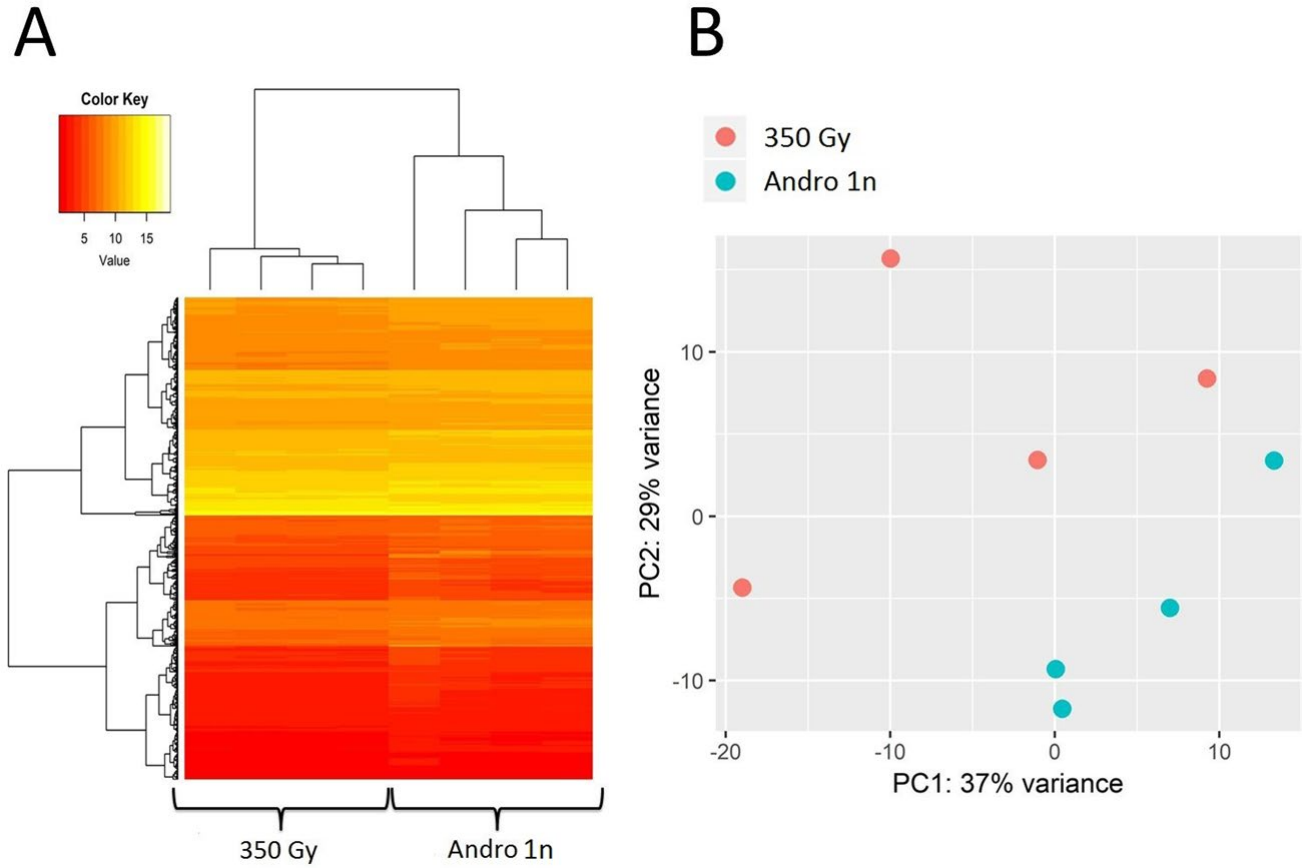
Changes in the transcriptome profile of rainbow trout eggs after androgenetic activation (Andro 1n) of irradiated (350 Gy) eggs. (**A**) Unsupervised hierarchical clustering of expression profiles based on probes with expression that differed significantly between groups (padj < 0.05). *An Euclidean distance and average hierarchical clustering was applied to the normalized genes expression levels*. (**B**) Principal component analysis of expression profiles for irradiated eggs before (350 Gy) and after fertilization (androgenetic activation) (Andro 1n).

**Figure 4:**
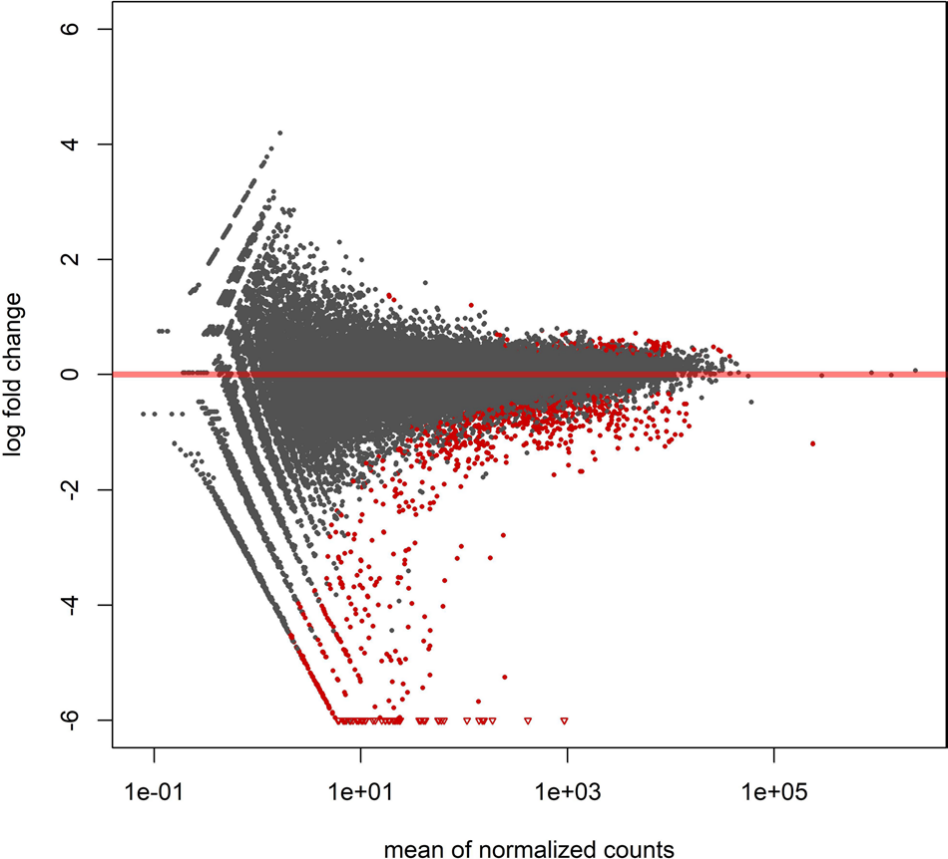
MA plot to compare expression levels between irradiated (350 Gy) eggs before and after fertilization. The plot presents mean expression levels for individual genes and their log_2_ fold-change between irradiated (350 Gy) eggs before and after fertilization (androgenetic activation). Red dots mark genes significantly differentially expressed between the groups (adjp < 0.05). Red triangles mark observations with outlying log_2_ fold-changes which would be difficult to plot on zoomed graph.

The hierarchical clustering and principal component analysis of the mRNA expression profiles showed that most of the expression variation in non-irradiated eggs and irradiated but not fertilized eggs resulted from the inter-individual variation (Supplementary File 3). However, clear differences in the expression profiles were observed between non-fertilized and fertilized irradiated eggs (Figs 3, 4; Supplementary File 3). The significant differences in the expression level were observed for 783 transcripts (belonging to 445 different genes), 687 (87.7%) of which were downregulated in the irradiated eggs after insemination (Supplementary File 4). On the other hand, transcriptome profiles of the fertilized non-irradiated eggs and fertilized irradiated eggs did not differ significantly (Supplementary File 5).

The altered transcripts observed in the fertilized irradiated eggs were associated with various biological processes, molecular functions and cellular components (Supplementary Table 3). Downregulated genes were mainly associated with metabolic and cellular processes, their regulation and response to stimuli, while the up-regulated genes were connected with e.g. RNA and mRNA processing, gene expression and macromolecule metabolic processes (Supplementary Table 3, Supplementary Files 6, 7).

Alterations in miRNAome profiles were also assessed in irradiated eggs before and after fertilization (Supplementary Files 2, 8). Four hours after insemination, changes in the microRNA profile of one-cell androgenetic embryos were observed, including upregulation of many miRNAs and their isomiRs (145) (Supplementary Files 8, 9, 10). On the pointwise level (p < 0.05), statistically significant differential expression was observed for 40 known and eight potentially novel microRNAs (Supplementary File 2). Most (68.75%) of these microRNAs were found to be upregulated (Supplementary File 2). For some microRNAs including ssa-miR-126-3p, ssa-miR-21a-5p, ssa-miR-21b-5, ssa-miR-146d-5p, ssa-miR-148a-3p, we observed both up- and down-regulation depending on their isomiRs (Table 2; Supplementary File 2).

Known microRNAs, which were differentially expressed after activation of the irradiated eggs enriched a wide range of biological processes. The most significant and interesting were those associated with the cellular nitrogen compound metabolic process (GO:0034641), gene expression (GO:0010467), the cellular protein modification process (GO:0006464), the response to stress (GO:0006950) and mRNA (GO:0016071) and RNA (GO:0016070) metabolic processes. The genes had molecular functions connected mainly with RNA binding (GO:0003723), cytoskeletal protein binding (GO:0008092), transcription factor binding (GO:0008134) and androgen receptor binding (GO:0050681). The differentially expressed (DE) microRNAs were also associated with cellular components, e.g. organelles (GO:0043226), cytosol (GO:0005829) and nucleoplasm (GO:0005654) (Supplementary File 11).

### qPCR verification of mRNA-Seq results

*EGR1* and *IER2* genes that had been significantly upregulated after irradiation of eggs with both radiation doses were selected for qPCR validation. The primers for amplification reactions were designed to capture all transcript variants of these genes (as specified in the applied reference transcriptome). The initial PCR analysis (performed to verify the specificity of primers) showed week or virtually no expression of the analyzed genes in the non-irradiated eggs (control samples), while clear amplification products were detected in eggs irradiated with 175 Gy and 350 Gy (Supplementary File 12). The performed qPCR analysis showed the presence of transcripts of the studied genes in eggs from all examined groups, however, clearly higher levels of transcripts were found in the irradiated eggs (Supplementary Tables 5 and 6). The correlation analysis of the expression levels obtained with the use of RNA-Seq and qPCR methods showed high concordance of the results in the case of the *EGR1* gene, where the correlation coefficient for individual samples exceeded 0.74. The correlation coefficient observed for the mean expression in the studied groups was also high for *EGR1* and exceeded 0.98. In the case of the *IER2* gene, the per-sample expression showed poor concordance between both molecular methods, however, the expression averaged per the studied groups nicely correlated between RNA-seq and qPCR methods, with Pearson’s coefficient of 0.920. In general, qPCR showed lower dynamics of expression changes compared to RNA-Seq, with fold change values clearly lower than in RNA-Seq. Nevertheless, the direction of changes and the relative size of changes among the studied groups were similar for both methods applied in the present research (Supplementary Tables 5 and 6).

## Discussion

The high concentration and integrity of RNAs extracted from irradiated eggs confirmed that the dose of 350 Gy applied to damage rainbow trout maternal chromosomes does not necessarily have to compromise the quality and functionality of the maternal RNA. The radiation resistance of the maternal RNA was confirmed in the loach (*Misgurnus fossilis*), whose newly fertilized eggs irradiated with 6–20 kR (60–200 Gy) were developing efficiently until the blastula stage^14^. Nevertheless, the dose of 60–100 kR (600–1000 Gy) arrested the development of loach embryos just after irradiation^14^. In the rainbow trout, radiation doses from 30 to 50 kR (300–500 Gy) were assessed as equally effective for inactivation of the maternal nuclear genome, while doses higher than 65 kR (650 Gy) exhibited a lethal effect^15,16^.

As the maternal mRNA deposited in eggs during oocyte maturation is repressed until egg activation, elevated levels of RNA observed in the irradiated rainbow trout eggs imply X-rays induced expression of the maternal genes. The radiation-induced gene expression was confirmed in many normal and tumor cells treated with X-ray doses much lower than those used to enucleate fish eggs^17,18^. In the present research, upregulated expression levels of transcripts of the immediate early response 2 gene (*IER2*) and the early growth response 1 gene *(EGR1*) were observed in eggs irradiated with 175 Gy, which suggests that the expression of these genes was activated quite early during the exposure to X-rays, before the functional damage of the irradiated genome. *IER2* and *EGR1* are members of “immediate early genes” that are rapidly activated by diverse extracellular stimuli including ionizing radiation^17^. The promoter of the early growth response gene (*EGR1*) was previously described to be activated by ionizing radiation in malignant cells^19^. In fish, transcription of the *IER2* gene is observed in the brain^20^, while the *EGR1* gene is expressed in the whole embryonic body^21^. As these genes are naturally expressed after the mid-blastula transition, their activity observed in the irradiated eggs confirmed that both genes were transcriptionally activated in response to irradiation.

The amount of RNA in the irradiated eggs decreased significantly after insemination (androgenesis) (p < 0.0001), which corresponded very well with downregulations of multiple (about 700) maternal transcripts that were also observed in inseminated irradiated eggs (Figs 2 and 3). Interestingly, no significant difference was observed between the transcriptome profiles of fertilized non-irradiated eggs and fertilized irradiated eggs (Supplementary File 5). Since a similar pattern of alterations in the RNA abundance was observed in fertilized non-irradiated and fertilized irradiated eggs, it is tempting to assume that the reduced level of mRNA observed in the fertilized irradiated rainbow trout eggs resulted from natural clearance of some of the maternal RNAs rather than from the irradiation process. Our results correspond well with what has been discovered in zebrafish, where most of the maternal RNAs are preferentially cleared after the activation of the zebrafish zygotic genome^22,23^, however, some cohorts of maternal transcripts are degraded just after egg fertilization^24,25^. In *Drosophila*, more than 20% of the maternal mRNAs (>1600) are eliminated upon egg activation^26^. The majority of mouse maternal mRNAs are cleared before zygotic transcription^27^. Analysis of the *C. elegans* transcriptome during the oocyte-to-embryo transition showed that thousands of maternal mRNAs are degraded before the 1-cell stage^28^. In the sea urchin, early degradation of maternal transcripts occurred 4 h after fertilization and before activation of the zygotic genome^29^.

Several genes involved mostly in the RNA processing, gene expression and nucleic acid metabolic processes were found to be upregulated in activated irradiated eggs. Recent studies in zebrafish also showed an increase in some of the mRNAs before activation of the zygotic genome^24,25^. Nevertheless, further experiments using the RNA-seq approach suggested that post-transcriptional regulation of maternal mRNA, including maturation, polyadenylation and de-adenylation rather than *de novo* transcription caused an increase in the mRNA level before maternal to zygotic transition (MZT)^25,30^. To be translated, transcripts need to acquire a polyA tail that regulates the rate of translation. The polyA tales can also be removed and re-acquired in cytoplasm during a process called cytoplasmic polyadenylation. Some of the mRNAs are polyadenylated and translated just after the transportation to the cytoplasm, while other mRNAs are deadenylated in the cytoplasm and stored there to be translated later^25,31^. The amount of RNA with polyA tails fluctuates upon egg activation^31^. In zebrafish, about 70% of the maternal transcripts exhibited increased abundance due to cytoplasmic polyadenylation between the 1-cell and 16-cell stage^25^. Moreover, it is assumed that the upregulation of maternal transcripts results from the failure of transcriptional activation of maternal mRNA degradation components^30^.

MicroRNAs (miRNAs) are small, non-coding RNAs that regulate the post-transcriptional expression of target mRNAs, mostly by their repression^32^. Thus, the analysis of miRNAs in irradiated fish eggs may shed a new light on the process of cellular responses to IR. Profiles of miRNAs in eggs and early embryos have been provided for a few fish species, including those considered as models in biomedical studies^33^ and commercially important species (aquaculture)^34,35^. To our knowledge, this is the first approach to assess IR-induced alterations in the abundance of miRNAs in fish eggs during induction of the androgenetic development. On the genome-wide level, changes in the trout egg miRNAs in response to IR were insignificant and concerned a relatively small number of transcripts. The majority of miRNAs, the expression levels of which changed during irradiation, were repressed (Supplementary File 2). This observation suggests the predominance of IR-induced degradation of miRNAs over *de novo* IR-induced activation of the miRNAs expression. The reduced level of ssa-miR-10b-5p and ssa-miR-21b-5p corresponded to the increased abundance of their target mRNAs (*IER2* and *EGR1*) observed in eggs irradiated with a lower IR dose.

The subtle increase in the miRNA abundance, observed in fertilized irradiated rainbow trout eggs, was accompanied by a noticeable downregulation of multiple gene transcripts (Supplementary File 2). This mirrored the nature of miRNAs that regulate the post-transcriptional expression of target mRNAs mostly by their repression. Members of the let-7 family, ssa-miR-133a-3p, ssa-miR-206-3p, ssa-miR-148-3p, ssa-miR-202-3p, ssa-miR-126-3p, ssa-miR-26b-5p were found to be the most altered miRNAs observed in fertilized irradiated rainbow trout eggs. Increased levels of let-7 and miR-148 were also observed in irradiated human tumor cells^36–38^ (Table 2).

Differential regulation of miRNAs in response to IR includes induction and repression of the miRNAs expression. Comparison of miRNA expression profiles in irradiated human cells and rainbow trout eggs revealed a similar pattern of response to IR, including increased expression of miR-22 and the reduced level of miR-30 (^38^, present research; Table 2).

Multiple variations in the miRNA sequences, known as isomiRs, were observed in irradiated and inseminated eggs of the rainbow trout. It is likely that miRNAs length variation occurs during the modification of the miRNA precursor by the Drosha/Dicer mechanism^39^ or is a result of different allelic forms and/or paralogous genes. In the present research, isomiRs of ssa-miR-7b-5p, ssa-miR-126-3p, ssa-miR-21a-5p, ssa-miR-21b-5, ssa-miR-146d-5p, ssa-miR-148a-3p, among others were found to be differentially expressed after irradiation. The varied response of different variants of miRNAs to IR suggested that isomiRs may regulate different target genes, which has been confirmed in other research^40^.

## Material and Methods

### Fish broodstock and gamete collection

Gametes were collected from 3- and 4-year-old individuals of spring spawning rainbow trout (*Oncorhynchus mykiss* Walbaum 1792) coming from the outbred selection strain (Rutki strain) cultured at the Department of Salmonid Research, Inland Fisheries Institute in Olsztyn, Rutki, Poland. Rainbow trout from the Rutki strain exhibit chromosomal polymorphism of the Robertsonian type and their diploid (2n) chromosome number ranges from 59 to 62, whereas the chromosome arm number value (FN) is stable and equals 104^41^.

Before manipulations, selected specimens were anesthetized using Propiscin (etomidate, IRŚ, Poland) at a dose of 0.5 ml l^−1^ of water. Eggs from four females were stripped and collected into separate polyethylene (PE) bowls. Eggs from each female (c. 3700) were then divided into three batches to produce haploid androgenetic (Andro 1n) (A), gynogenetic (Gyno 1n) (G) and diploid control (C) groups and transferred to separate plastic containers with covers, submerged in the ovarian fluid and kept at 8–10 °C for further treatment. Milt from four males was collected into the separate PE containers. To confirm the sperm motility, 1 μl of semen with an addition of 49 μl of the sperm activating medium (SAM; 154 mM NaCl and 1 mM Ca^2+^, buffered to pH 9.0 with 20 mM Tris + 30 mM glycine)^42^ was placed on a glass slide and analyzed under a microscope (Nikon Eclipse E 2000). Sperm from all striped males showed high (>80%) motility. Sperm samples were refrigerated at 2 °C for further use.

### Inactivation of the parental nuclear DNA

Batches of eggs to be used for androgenesis were placed in a cooler box and transported to the Clinic of Oncology and Radiotherapy, University Clinical Center, Medical University of Gdansk. A standard irradiation method was used to inactivate the nuclear DNA in rainbow trout eggs^9,43^. A TrueBeam linear accelerator (Varian Medical Systems, Palo Alto, CA, USA) was used to irradiate eggs [350 Gy of X-rays (6 Gy/min)]. Eggs were irradiated from two opposed fields (from top and from bottom) with a dose of 175 Gy from each field. The distance from the radiation source was 98.7 cm. During irradiation that lasted a. 66 min, containers with eggs immersed in ovarian fluid that formed a layer of 26 mm were placed on a PE tray with crushed ice.

Sperm from four rainbow trout males was pooled and diluted in the rainbow trout seminal plasma (40×). A 60 ml glass beaker (50 mm diameter, 30 mm height) with 15.375 ml of diluted sperm (depth of diluted sperm: 7.8 mm) was placed on a magnetic stirrer and exposed to the UV-C light source (Phillips TUW 30 Watt UV bulb) for 11 min. The distance between the surface of the magnetic stirrer and the UV lamp was 20 cm. The UV intensity and UV dose were 2075 μW/cm^2^ and 1369.5 J/m^2^, respectively. During the irradiation, the diluted sperm was mixed using the magnetic stirrer (1400 × g). The irradiated sperm was used for the gynogenetic egg activation immediately after the UV exposure.

### Induction of haploid androgenesis and gynogenesis

One hour after irradiation, batches of irradiated eggs were separately inseminated with spermatozoa from each male in the presence of SAM^43^ to provide androgenetic haploids (Andro 1n)^11,43^. Portions of diluted and UV-irradiated sperm were added separately to batches of non-irradiated eggs from four females (~150,000 spermatozoids per egg) and poured over with SAM to induce gynogenetic development (Gyno 1n). To provide diploid control groups (C), non-irradiated eggs were inseminated separately with milt from each male.

Fertilized eggs were placed in a hatching apparatus and incubated at 9 °C at the Department of Salmonid Research, Rutki. The survival of normal diploid and haploid androgenetic and gynogenetic embryos were calculated 27 days post fertilization (dpf) (eyed stage) and 57 dpf (swim-up stage).

For the transcriptome analysis, portions of about 50 non-activated eggs from each batch/female were taken and transferred into 50 ml plastic falcons with RNA-later solution (Sigma Aldrich) before irradiation (non-irradiated eggs) (1), after irradiation with 175 Gy (2), and after irradiation with 350 Gy of X rays (3). Moreover, fertilized (activated) irradiated eggs (Andro 1n) (n = 50) (4) and activated non-irradiated eggs (Gyno 1n) (n = 50) (5) from each female were taken and submerged in the RNA-later solution 4 h after insemination (5 h after egg irradiation) (4).

### Cytogenetic examination of embryos

Androgenetic and normal diploid rainbow trout eyed embryos were cytogenetically studied using the *in vivo* technique^7^ to confirm their haploid state. For the purpose of visualization, chromosomes were stained with 4′,6-diamidino-2-phenylindole DAPI (Vector, Burlingame, USA) and examined under a Zeiss Axio Imager A1 microscope equipped with a fluorescent lamp and a digital camera (Applied Spectral Imaging, Galilee, Israel). Images were captured and processed using the Band View/FISH View software (Applied Spectral Imaging).

### RNA quantity and degradation analysis

Eggs preserved in RNA-later solution were incubated overnight in a refrigerator (4 °C) and finally stored at −80 °C for RNA purification. To assess the relative RNA yield in eggs from each female, four eggs of similar size from each batch [non-irradiated eggs, eggs irradiated with 175 Gy, eggs irradiated with 350 Gy, fertilzied irradiated eggs (Andro 1n), and gynogenetically activated non-irradiated eggs (Gyno 1n)] were thawed on ice and homogenized in TRIzol Reagent (Thermo Fisher Scientific) using a manual method to avoid homogenization-induced mRNA degradation. RNA was purified using the modified TRIzol procedure established at the Igor Babiak laboratory, University of Nordland, Norway. The obtained RNA was suspended in the equal volumes and quantified using three different methods: (i) the spectrophotometric method – with a NanoDrop2000 spectrophotometer (Thermo Fisher Scientific), (ii) the fluorometric method – using RNA binding dye and a Qubit 2.0 Fluorometer (Thermo Fisher Scientific) and (iii) the electrophoretic method with RNA-binding dye using the Agilent 2200 TapeStation system (RNA screen tapes). For each quantification, the RNA purification was carried out in separate rounds of preparation to account for bias associated with egg size and random sampling. Separate rounds of RNA purification also allowed to account for the effect of some potential small differences in the course of the manual RNA isolation protocol. An Agilent’s RIN (RNA Integrity Number) algorithm was also used to compare the RNA quality/integrity between separate treatments. The algorithm analyzes the ratio of 18S and 28S rRNA and the whole electrophoretic trace of a sample, which indicates the presence or absence of degradation signs. The assigned RIN is independent of the sample concentration and assigns a value of 1 to 10 to an electropherogram, with 10 being the highest quality RNA. RIN is standardized and can be used for comparative studies^44^.

To evaluate the impact of irradiation on the quantity and quality of the maternal mRNA from rainbow trout eggs, the analyzed parameters were compared between different groups using the paired and unpaired t-test.

### Whole transcriptome sequencing and data analysis

To provide the highest RNA purity needed for sequencing, the total RNA was additionally purified before the library construction, applying Agencourt RNAClean XP beads (Beckman Coulter). 800 ng of purified RNA was used for the TruSeq RNA Sample Prep v2 kit (Illumina). Library construction process included mRNA selection, fragmentation, cDNA synthesis, end repair, adenylation, indexed adapter ligation and amplification and it was followed by quantitative (Qubit, Thermo Fisher Scientific) and qualitative (Agilent TapeStation 2200) assessment. Quality controlled and normalized libraries were ultimately sequenced in a single 50-bp run (1 × 50 bp) using the HiScanSQ system with application of the TruSeq SBSv3 Sequencing kit (Illumina) to achieve approx. 25 million reads per sample.

Achieved demultiplexed raw reads were controlled for quality using the FastQC software and filtered using the Flexbar software^44^ in order to trim a random sequence of adapters and remove low quality reads. The obtained high quality reads were mapped against the newest accessible rainbow trout transcriptome atlas (encompassing 44 990 transcripts; downloaded from http://www.animalgenome.org/repository/pub/MTSU2014.1218/, using Bowtie software^45^, the set for unlimited multi-mappings (−a). The mapped reads were studied using the eXpress software (http://bio.math.berkeley.edu/eXpress/overview.html^46^. Roberts and Pachter, 2012), which was designed to estimate the number of transcripts in multi-isoform genes and was able to resolve multi-mappings of reads across gene families. As the software does not need a reference genome, it may be used with *de novo* transcriptome assemblies. The implemented model is based on the formerly described probabilistic models established for RNA-Seq.^47^. Nevertheless, it is also appropriate to other settings where target sequences are sampled and contain values for fragment size distributions, errors in reads and a fragment sequence bias^48^. The achieved assessed rounded effective read counts for separate transcripts and samples were calculated using DESeq2^49^. The data analysis steps followed guidelines presented by Roberts and Pachter^46^ in the eXpress software guide.

The functional annotation and gene ontology (GO) analysis of differentially expressed transcripts was performed with the Blast2GO software^50^.

### miRNA library preparation, sequencing and data analysis

For miRNA analysis, RNA was purified using an in-house modified procedure with the use of the Direct-zol RNA Mini Prep kit (Zymo Research). In brief, eggs were manually homogenized in TRIzol solution and subjected to phase-separation with chloroform according to a protocol established at Igor Babiak Laboratory (University of Nordland, Bodo, Norway)^34^. The upper phase and 99.8% EtOH were mixed (1:1) and from this point, the Direct-zol RNA Mini Prep protocol was followed. The extracted RNA was assessed in terms of its quantity and quality, with the use of a NanoDrop 2000 spectrophotometer (Thermo Fisher Scientific) and a TapeStation 2200 instrument (Agilent), respectively. The amount of 500 ng of RNA was used to construct microRNA libraries using the NEBNext Multiplex Small RNA Library Prep Set for Illumina (New England Biolabs) and following the standard protocol with the multiplexing option (3′ adaptor ligation, Reverse Transcription Primer ligation, 5′ adaptor ligation, PCR amplification, PAGE gel purification and EtOH precipitation). The libraries were subjected to concentration measurements using a Qubit 2.0 Fluorometer (Thermo Fisher Scientific) and size measurements with a 2200 TapeStation instrument (Agilent). The normalized miRNA libraries were finally sequenced with the PhiX control library (Illumina) in a single 50-bp run on the HiScanSQ system using the TruSeq SBSv3 Sequencing kit (Illumina).

The acquired reads were controlled for quality using the FastQC software^51^ and then analyzed with the use of UEA sRNA Workbench V3.2^52^. First, 3′ adaptor sequences were trimmed off and the sequences were filtered according to the following parameters: 17–35 nt in length, minimum abundance of at least 6 supporting reads, tRNA and rRNA sequences discarded from the data. The microRNA sequences were identified with reference to the rainbow trout genome (https://www.genoscope.cns.fr/trout/) and miRBase v21.0 Atlantic salmon *Salmo salar* homologues^53,54^, applying the miRCat tool with default parameters for animals with the exception of minimum length (17 nt), maximum length (25 nt) and minimum abundance (6). In addition, potentially novel miRNA sequences were checked for the presence of other non-coding RNA species, using the RNAcentral database (the RNAcentral Consortium, 2017). In the last step, the microRNA length and sequence variants (isomiRs) were identified using the isomiR-SEA software^55^ with the default settings.

The differential expression analysis of the identified miRNAs was performed using the DESeq2 software^56^ following the software manual. The miRNAs showing statistically significant differences between the tested samples (p ≤ 0.05) were selected for further analysis.

The differentially expressed miRNAs were analyzed using the mirPath v.3 DIANA Tools web application^57^ to identify their target genes and enriched pathways. We chose Tarbase v 8.0 and microT-CDS bases as reference databases of miRNA target genes, while the GO database was employed for the pathway enrichment analysis. As rainbow trout microRNAs are not present in the miRBase (21.0), human and zebrafish *Danio rerio* homologues deposited in miRBase (21.0) were used.

### qPCR validation of RNA-Seq results

For qPCR validation, two genes: the immediate early response 2 (*IER2*) gene and the early growth response 1 (*EGR1*) gene, differentially expressed in the irradiated eggs, were selected. The primers for the amplification of the gene fragments (Supplementary Table 4) were designed with the use of the Primer3 software within the transcript regions present in all splicing variants found in the employed version of the transcriptome. The *EF1-α* (Translation elongation factor 1-alpha) gene was selected as an endogenous control and amplified using primers previously described by Bland *et al*.^58^. This gene had a stable transcript level in all analyzed groups in the present study as well as it was previously shown to be an useful endogenous control in several fish species^58^. The purified total RNA was transcribed into cDNA using the High-Capacity RNA-to-cDNA™ Kit (Thermo Fisher Scientific, MA, USA). Expression levels were determined with the QuantStudio 7 Flex System (Thermo Fisher Scientific, Waltham, MA, USA) using PowerUp SYBR™ Green Master Mix (Thermo Fisher Scientific, Waltham, MA, USA) and the standard manufacturer protocol. The specificity of primers was confirmed by melting curve analysis and agarose gel electrophoresis of PCR products. The qPCR reactions with cDNA samples from non-irradiated eggs, eggs irradiated with 175 Gy and 350 Gy were conducted in triplicates with corresponding negative controls. The efficiency of the amplification reaction for different pairs of primers was determined using a standard curve method. The relative expression ratio was calculated according to Pfaffl^59^. Expression fold changes obtained from RNA-Seq and qPCR were compared. In addition, correlation coefficients between the results of RNA-Seq and qPCR were calculated for the expression levels established in individual samples as well as for the group means (Supplementary File 12).

### Approval (Ethical Committee for the Experiments on Animals)

This study was performed in compliance with the recommendations contained in the Polish Act of 21 January 2005 on Experiments on Animals (Dz. U. of 2005, No. 33, item 289). The protocol was approved by the Local Ethical Committee for the Experiments on Animals of the University of Gdansk, Poland (no. 28/2015). This article does not cover any studies with human participants, performed by any of the authors.

### Accordance

All methods applied in the present paper were carried out *in accordance with* the relevant guidelines and regulations.

## Supplementary information

Dataset 1

Dataset 3

Dataset 2

Dataset 4

Dataset 5

Dataset 6

Dataset 7

## Data Availability

The mRNA and miRNA sequencing reads were deposited in the SRA (Sequence Read Archive) database, under the accession number SRP156714.
