# Supplementary material for: Induced androgenetic development in rainbow trout and transcriptome analysis of irradiated eggs
Source: Sci Rep. 2019 May 30;9:8084. doi: 10.1038/s41598-019-44568-7 (PMC6542805; doi:10.1038/s41598-019-44568-7)
Supplement: Supplementary file 3 — Dataset 2 [file 41598_2019_44568_MOESM3_ESM.pdf]

Supplementary Files

Induced androgenetic development in rainbow trout and transcriptome analysis of irradiated eggs

Konrad Ocalewicz[1]*, Artur Gurgul[2], Klaudia Pawlina-Tyszko[2], Tomasz Szmatoła[2, 3], Igor Jasielczuk[2, 3], Monika Bugno-Poniewierska[4], Stefan Dobosz[5],

Supplementary File 1. Gene expression analysis of- rainbow trout non-irradiated eggs (control; ctr) and eggs irradiated with 175 Gy and 350 Gy. (**Excel**)

Supplementary File 2. The results of differential microRNA expression analysis between non-irradiated eggs (control; ctr) and eggs irradiated with 175 Gy and 350 Gy as well as inseminated irradiated (Andro 1n) eggs. *NA in the miRNA name column stands for potentially new, so far not annotated microRNAs. Statistically significant isomiRs were bolded.* **(Excel)**

Supplementary File 3. Differences in the transcription profile of eggs from different experimental groups based on 200 transcripts with the highest standard deviation among the analyzed samples.

A – Unsupervised hierarchical clustering of expression profiles for all study groups.

B – Principal component analysis of expression profiles for all study groups.

Sample labels are in accordance with Table 1.

Supplementary File 4. Upregulated and downregulated transcripts and their GO annotations observed in inseminated and irradiated (350 Gy) rainbow trout eggs (androgenesis). **(Excel)**

Supplementary File 6. Biological processes associated with genes that were upregulated in the fertilized irradiated (350 Gy) rainbow trout eggs.

Supplementary File 7. Biological processes associated with genes that were downregulated in the fertilized irradiated (350 Gy) rainbow trout eggs.

Supplementary File 8. Principal component analysis of miRNA expression profiles for irradiated (350 Gy) and irradiated and inseminated rainbow trout eggs (Andro 1n).

Supplementary File 9. Results of DESeq2 analysis of miRNA expression in the irradiated, and fertilized irradiated rainbow trout eggs

Supplementary File 110. Changes in the expression profile of miRNA in the rainbow trout irradiated (350) eggs after insemination. *An unsupervised hierarchical clustering of expression profiles based on probes with adjP<0.05.*

Supplementary File 11. GO annotations associated with differentially expressed miRNAs in fertilized irradiated (350 Gy) rainbow trout eggs based on human and *Danio rerio* homologs.

Supplementary File 12. Electrophoresis of PCR products (from primers specificity analysis) for the genes analyzed in non-irradiated eggs (CTR), eggs irradiated with 175 Gy and 350 Gy and randomly selected samples per each examined group.

Supplementary Table 1. Reads and mapping statistics.

Supplementary Table 2. Reads and miRNA mapping statistics.

Supplementary Table 3. Top ten of the most common biological processes, molecular functions and cellular components associated with transcripts that were up- or downregulated after insemination of the irradiated eggs.

Supplementary Table 4. Primers used for qPCR analysis performed to validate results of RNA-Seq.

Supplementary Table 5. Comparison of expression levels of *EGR1* gene detected by RNA-Seq and qPCR analysis in the non-irradiated eggs and eggs irradiated with 175 Gy and 350 Gy.

Supplementary Table 6. Comparison of expression levels of *IER2* gene detected by RNA-Seq and qPCR analysis in the non-irradiated eggs and eggs irradiated with 175 Gy and 350 Gy

Supplementary File 1. Gene expression analysis of- rainbow trout non-irradiated eggs (control; ctr) and eggs irradiated with 175 Gy and 350 Gy. (**Excel**)

Supplementary File 2. The results of differential microRNA expression analysis between non-irradiated eggs (control; ctr) and eggs irradiated with 175 Gy and 350 Gy as well as inseminated irradiated (Andro 1n) eggs. *NA in the miRNA name column stands for potentially new, so far not annotated microRNAs. Statistically significant isomiRs were bolded.* **(Excel)**

Supplementary File 3. Differences in the transcription profile of eggs from different experimental groups based on 200 transcripts with the highest standard deviation among the analyzed samples.
A – Unsupervised hierarchical clustering of expression profiles for all study groups.

B – Principal component analysis of expression profiles for all study groups.

Sample labels are in accordance with Table 1.

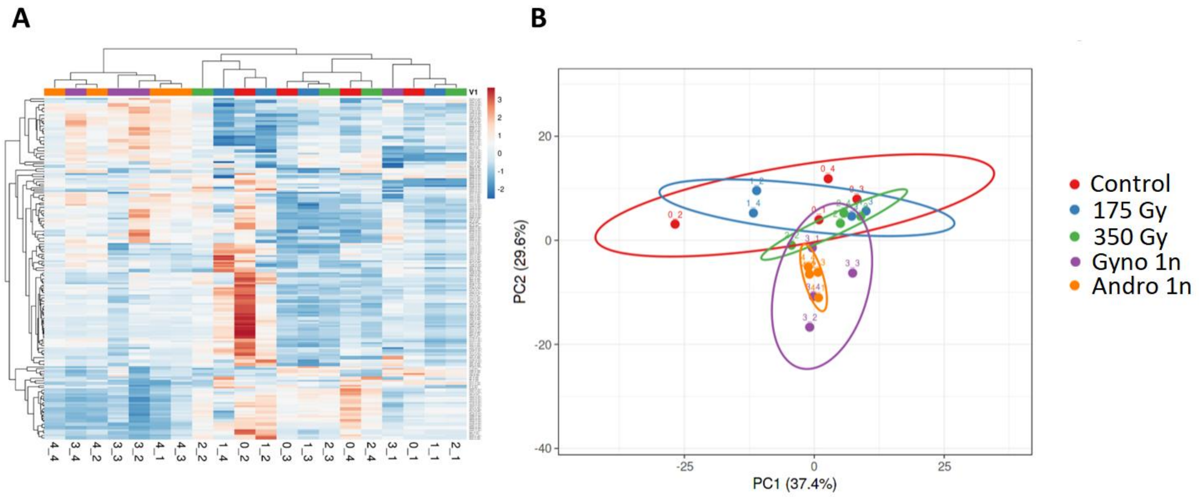

Supplementary File 4. Upregulated and downregulated transcripts and their GO annotations observed in inseminated irradiated (350 Gy) rainbow trout eggs (androgenesis). **(Excel)**

Supplementary File 5. MA plot to compare expression levels between activated irradiated and activated non-irradiated eggs.

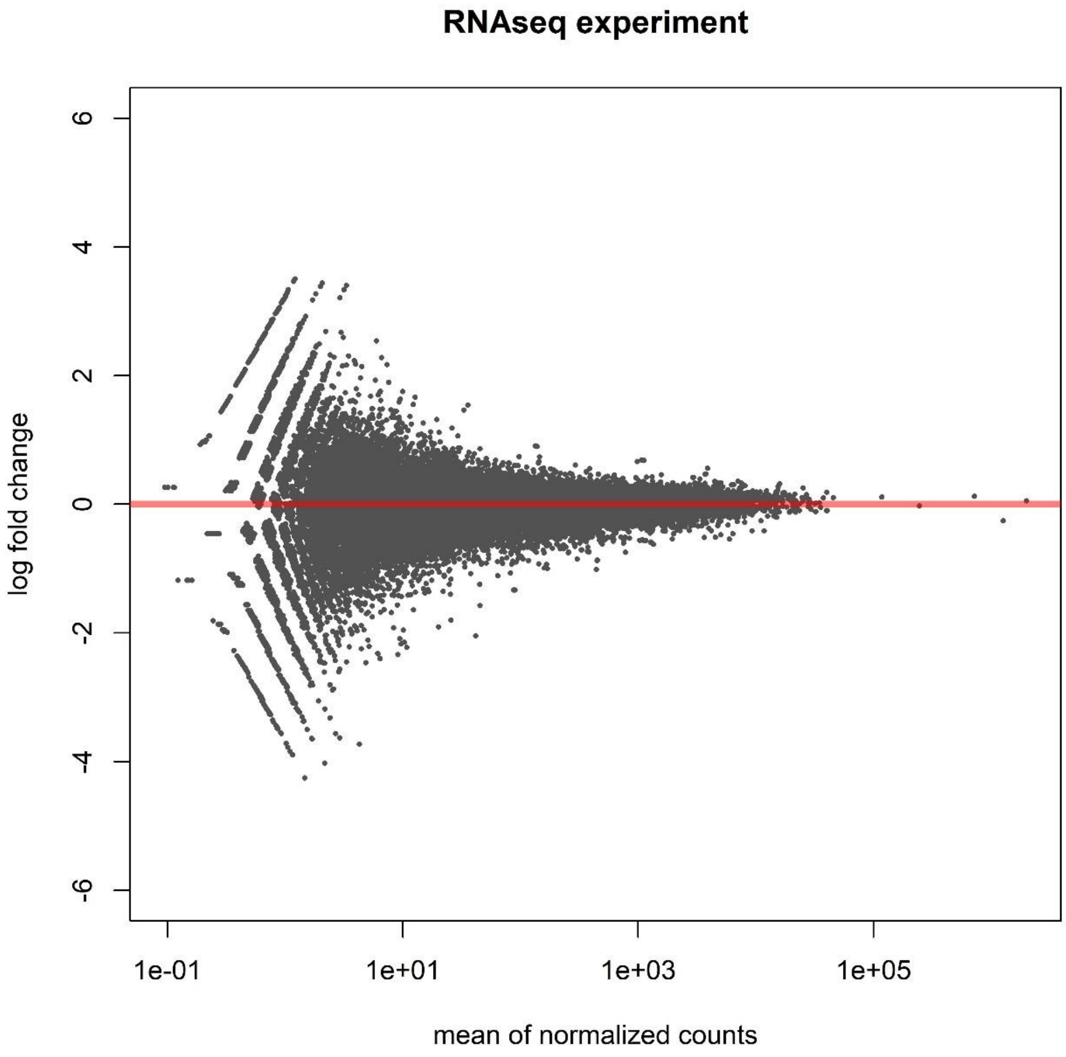

Supplementary File 6. Biological processes associated with genes that were upregulated in the fertilized irradiated (350 Gy) rainbow trout eggs.

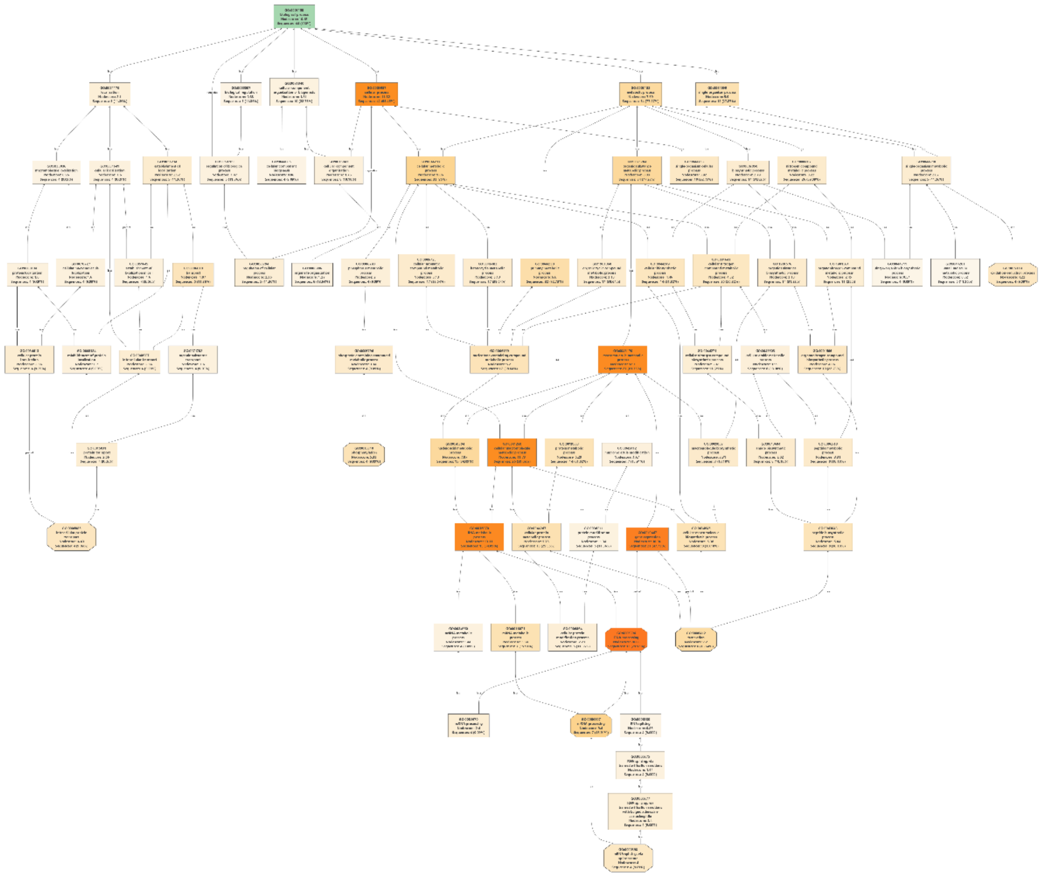

Supplementary File 7. Biological processes associated with genes that were downregulated in the fertilized irradiated (350 Gy) rainbow trout eggs.

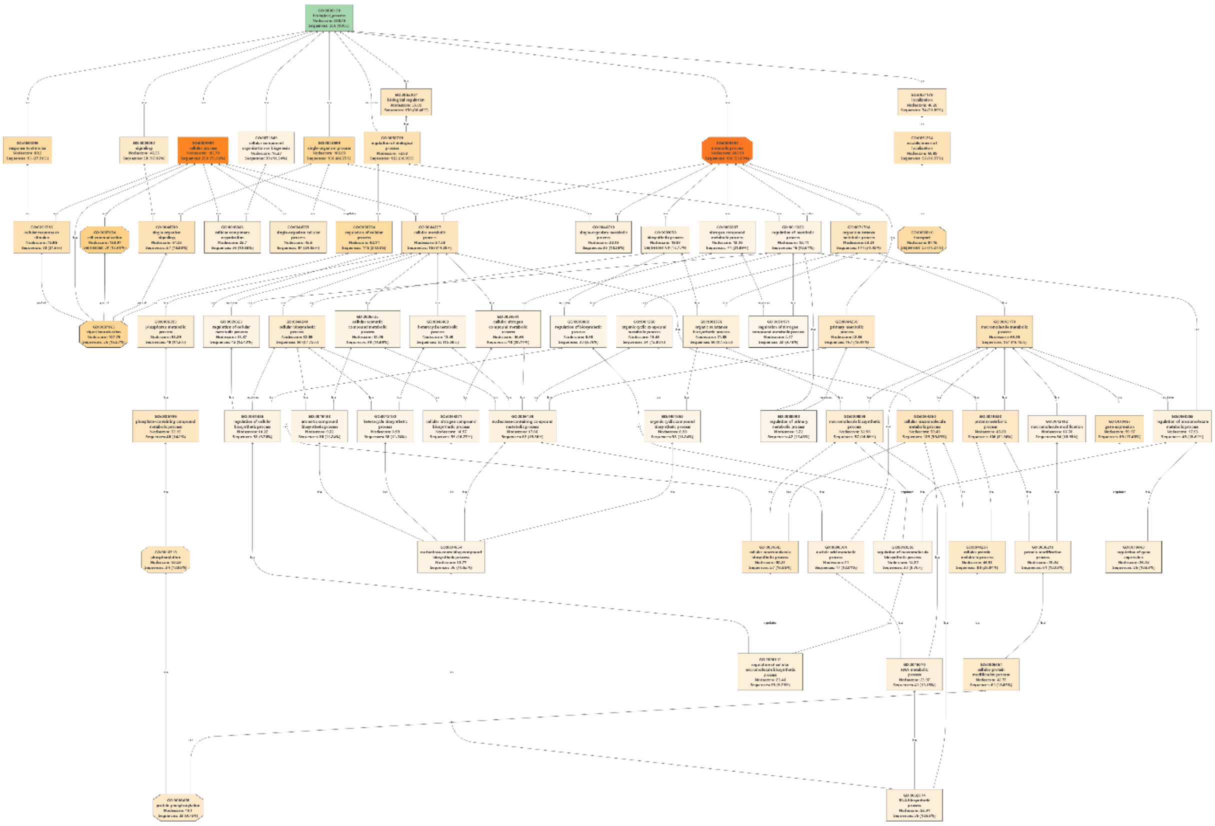

Supplementary File 8. Principal component analysis of miRNA expression profiles for irradiated (350 Gy) and fertilized irradiated rainbow trout eggs (Andro 1n).

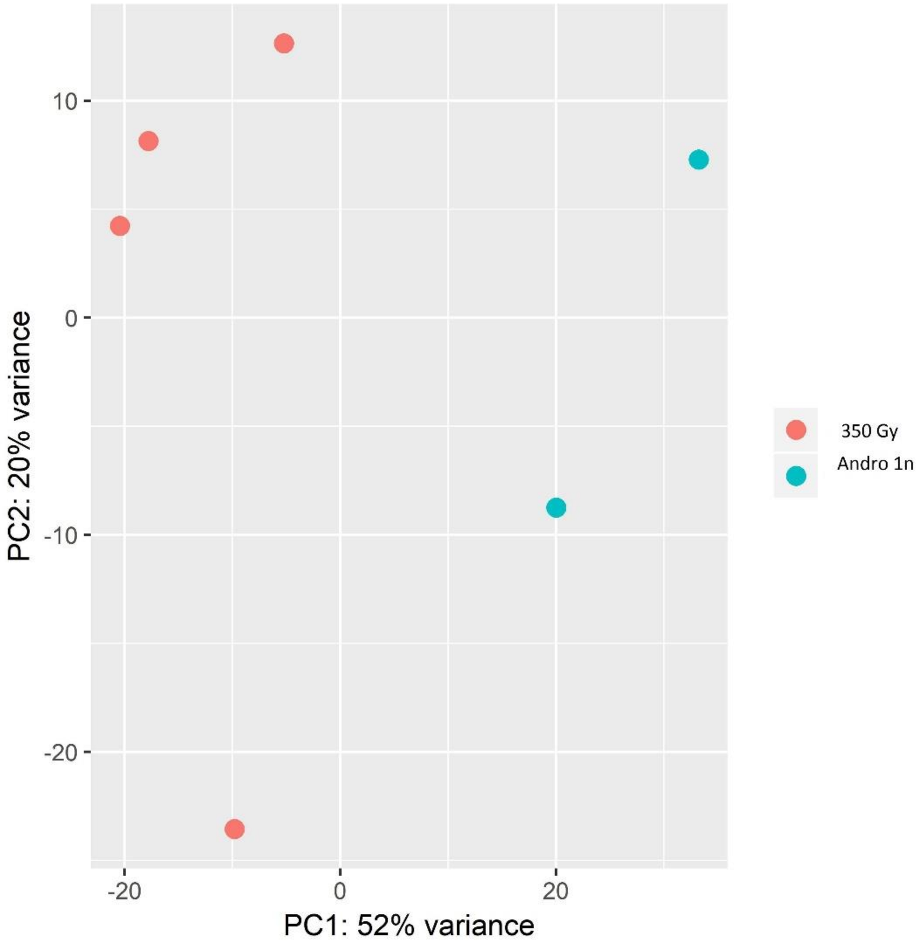

Supplementary File 9. Results of DESeq2 analysis of miRNA expression in the irradiated, and fertilized irradiated rainbow trout eggs

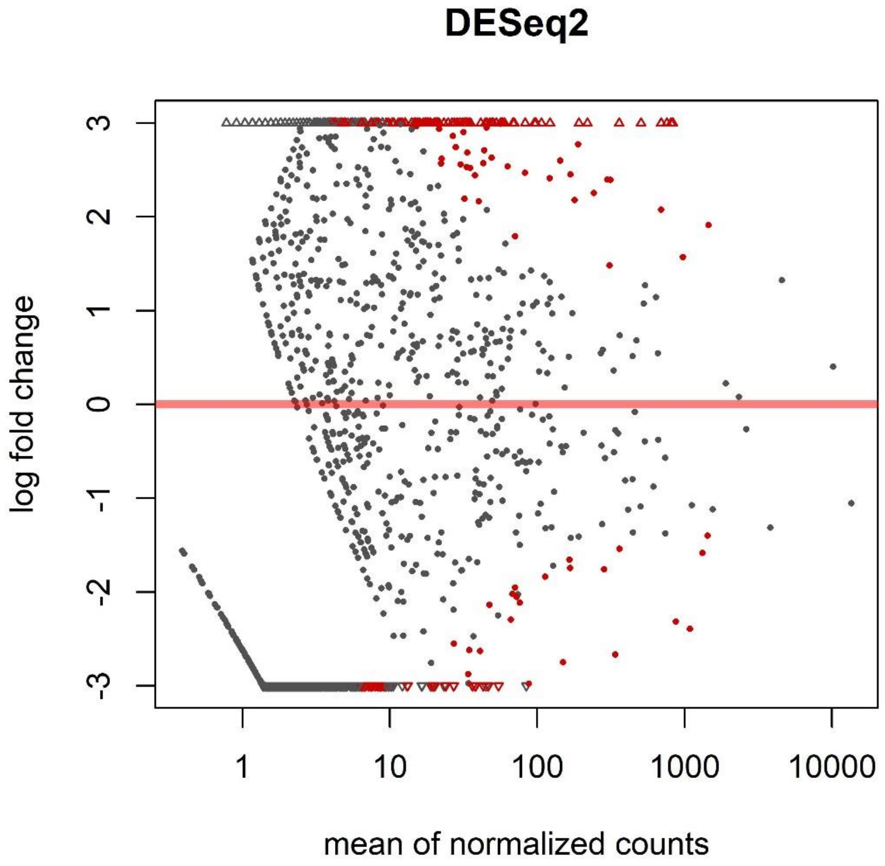

Supplementary File 10. Changes in the expression profile of miRNA in the rainbow trout irradiated (350) eggs after insemination. *An unsupervised hierarchical clustering of expression profiles based on probes with adjP<0.05.*

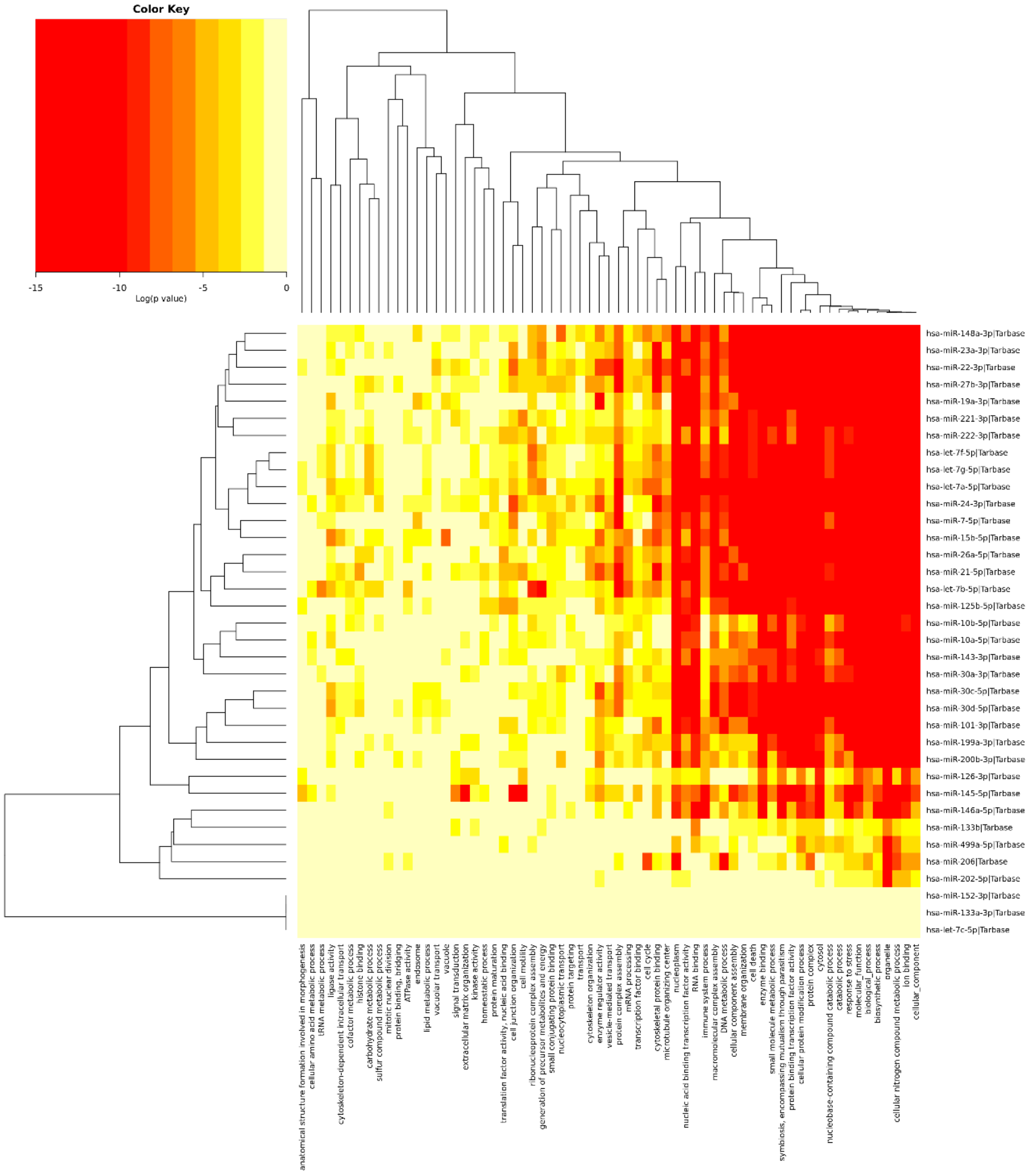

Supplementary File 11. GO annotations associated with differentially expressed miRNAs in irradiated (350 Gy) and inseminated rainbow trout eggs based on human and *Danio rerio* homologs (Excel).

Supplementary File 12. Electrophoresis of PCR products (from primers specificity analysis) for the genes analyzed in non-irradiated eggs (CTR), eggs irradiated with 175 Gy and 350 Gy and randomly selected samples per each examined group.

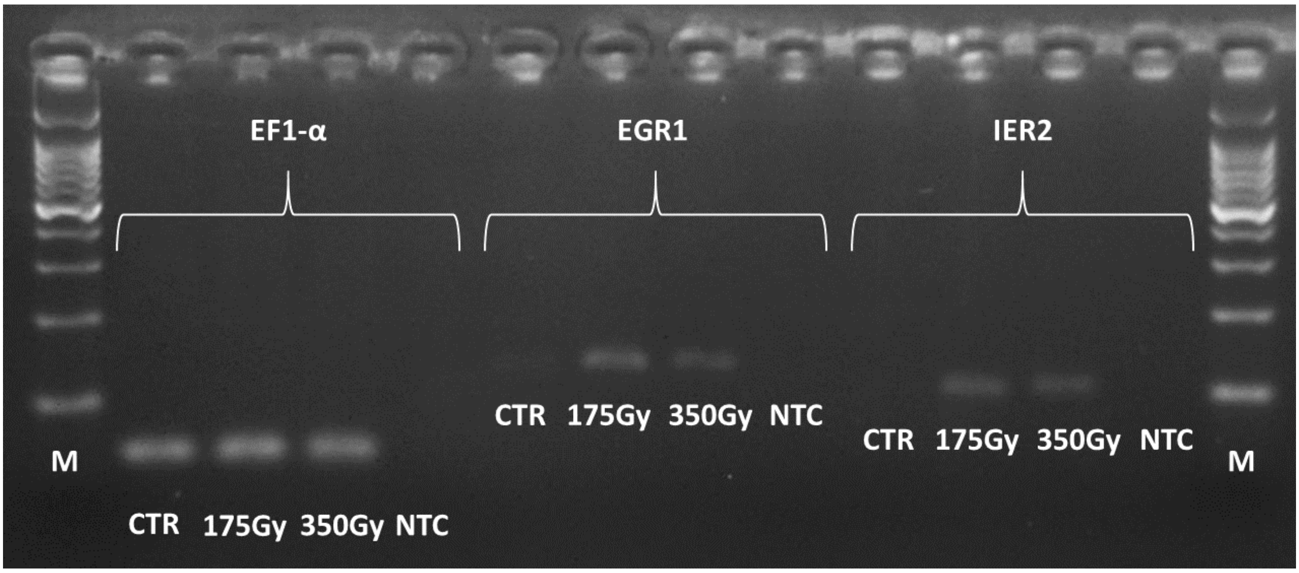

M- marker ladder (100bp); NTC- negative control

Supplementary Table 1. Reads and mapping statistics

Sample	Group	Number of reads after filtering	Number of reads with at least one reported alignment	Percentage of reads with at least one reported alignment	Number of expressed transcripts (normalize d read count >1)
0.1	Non-irradiated (Control)	23329582	14753627	63.24	31211
0.2		23234237	14895469	64.11	30680
0.3		32608418	21382212	65.57	29574
0.4		27542992	18078497	65.64	29079
Non-irradiated	Mean	26678807	17277451	64.64	30136
1.1	175 Gy	23192645	4602848	62.96	31510
1.2		21568842	13459830	62.4	31201
1.3		27438534	17660013	64.36	28773
1.4		20785915	13568851	65.28	30630
175 Gy	Mean	23246484	12322886	63.75	30529
2.1	350 Gy	42993938	26940396	62.66	30493
2.2		34911681	21387226	61.26	29875
2.3		23146317	14564747	62.92	31012
2.4		19246709	12260382	62.7	30304
350 Gy	Mean	30074661	18788188	62.385	30421
3.1	Gyno 1n	22173862	19291259	63.11	30568
3.2		16834737	14646221	62.48	29561
3.3		17129142	14902353	62.84	29650
3.4		16326421	14203986	63.24	29560
Gyno 1n	Mean	18116041	15760955	62.92	29835
4.1	Andro 1n	30929804	18968280	61.33	28412
4.2		30788592	19531872	63.44	28225
4.3		26891921	16645982	61.9	27992
4.4		26564821	16916886	63.68	27970
Andro 1n	Mean	28793785	18015755	62.59	28150
All	Mean	25381956	16433047	63.256	29814

Supplementary Table 2. Reads and miRNA mapping statistics

Sample	Group	Total reads						Distinct reads					
		input	filter by sequence length	filter low-complexity sequences	filter by min-abundance and max-abundance	filter invalid sequences	filter by t/rRNA (matches out)	input	filter by sequence length	filter low-complexity sequences	filter by min-abundance and max-abundance	filter invalid sequences	filter by t/rRNA (matches out)
0.1	Non-irradiated (Control)	8842583	4491067	4489638	3604482	3599703	3494851	1650697	693981	692808	31815	31689	30710
0.2		987429	529840	529755	372695	372360	359262	283375	128630	128552	5983	5964	5457
0.3		1841566	709777	709700	480538	480228	471505	499545	187555	187484	7208	7186	7062
0.4		15639226	7293510	7288367	5555607	5550468	5383871	2708541	1292721	1289159	60993	60844	59357
	Mean	6827701	3256048.5	3254365	2503330.5	2500689.75	2427372.25	1285539.5	575721.75	574500.75	26499.75	26420.75	25646.5
1.1	175 Gy	8812544	4519286	4517936	3645595	3640937	3539288	1668573	667191	666105	34752	34628	33903
1.2		2428258	1235978	1235751	942107	940963	920981	616156	240693	240479	9794	9746	9462
1.3		2927138	1298592	1298384	956287	955502	935324	734626	281530	281335	10911	10868	10668
1.4		5613304	3096971	3095876	2588100	2585405	2518165	943392	420619	419704	18114	18018	17543
	Mean	4945311	2537706.75	2536986.75	2033022.25	2030701.75	1978439.5	990686.75	402508.25	401905.75	18392.75	18315	17894
2.1	350 Gy	6565158	3082502	3081589	2393062	2390413	2338536	1339389	515770	515065	28116	28032	27407
2.2		1403489	729612	729557	607369	606957	521833	244862	100102	100051	7724	7699	7115
2.3		4505981	2186565	2186377	1722111	1720647	1683633	898519	366091	365911	18722	18656	18253
2.4		5108548	2798083	2796630	2227033	2224980	2173761	981105	453294	452178	20481	20402	19830

	Mean	4395794	2199190.5	2198538.25	1737393.75	1735749.25	1679440.75	865968.75	358814.25	358301.25	18760.75	18697.25	18151.25
4.2	Andro 1n	2461680	1383333	1383110	1006837	1005790	995121	583467	269127	268971	13639	13590	13199
4.4		1333031	815813	815688	640230	639613	629527	291748	137941	137835	5971	5948	5720
	Mean	1897355.5	1099573	1099399	823533.5	822701.5	812324	437607.5	203534	203403	9805	9769	9459.5
All	Mean	4890709.643	2440780.643	2439882.714	1910146.643	1908140.429	1854689.857	960285.3571	411088.9286	410402.6429	19587.35714	19519.28571	18977.57143

Supplementary Table 3. Top ten of the most common biological processes, molecular functions and cellular components associated with transcripts that were up- or downregulated after insemination of the irradiated eggs.

Upregulated				Downregulated			
Biological processes							
GO ID	GO Name	NS	#Seqs	GO ID	GO Name	NS	#Seqs
GO:0006396	RNA processing	20,08	13	GO:0008152	metabolic process	246,19	186
GO:0010467	gene expression	16,94	21	GO:0009987	cellular process	183,78	250
GO:0016070	RNA metabolic process	13,11	15	GO:0007154	cell communication	123,97	59
GO:0043170	macromolecule metabolic process	12,3	27	GO:0007165	signal transduction	107,75	56
GO:0044260	cellular macromolecule metabolic process	11,78	26	GO:0044699	single-organism process	106,03	158
GO:0009987	cellular process	11,32	42	GO:0050794	regulation of cellular process	92,11	115
GO:0006397	mRNA processing	9,4	7	GO:0050896	response to stimulus	88,5	93
GO:0044237	cellular metabolic process	9,06	33	GO:0006810	transport	87,16	55
GO:0071704	organic substance metabolic process	8,08	34	GO:0051716	cellular response to stimulus	72,96	73
GO:0090304	nucleic acid metabolic process	7,87	15	GO:0050789	regulation of biological process	72,63	122
Molecular functions							
GO:0005488	binding	37,34	52	GO:0005488	binding	540,4	313
GO:0003676	nucleic acid binding	33,25	23	GO:0003824	catalytic activity	247,43	149
GO:0003824	catalytic activity	31,73	26	GO:0005515	protein binding	174,47	144
GO:1901363	heterocyclic compound binding	30,34	32	GO:0046872	metal ion binding	158	98
GO:0097159	organic cyclic compound binding	30,34	32	GO:0016787	hydrolase activity	106,17	82
GO:0005515	protein binding	24,18	21	GO:0003676	nucleic acid binding	103,58	66
GO:0000166	nucleotide binding	21,56	20	GO:0097159	organic cyclic compound binding	103,02	133

GO:1901265	nucleoside phosphate binding	16,32	20	GO:1901363	heterocyclic compound binding	102,44	131
GO:0036094	small molecule binding	13,63	21	GO:0043169	cation binding	94,8	98
GO:0003723	RNA binding	11,72	11	GO:0046914	transition metal ion binding	81	51
GO:0005524	ATP binding	8	8	GO:0000166	nucleotide binding	72,79	63
Cellular components							
GO:0005622	intracellular	51,47	36	GO:0005623	cell	305,46	162
GO:0044464	cell part	35,82	36	GO:0005622	intracellular	298,99	143
GO:0005575	cellular component	33,12	39	GO:0044464	cell part	239,1	160
GO:0044424	intracellular part	22,31	29	GO:0016020	membrane	218,86	164
GO:0005623	cell	21,55	36	GO:0044424	intracellular part	197,22	127
GO:0030529	intracellular ribonucleoprotein complex	16,26	13	GO:0005737	cytoplasm	142,6	77
GO:0005634	nucleus	14,8	13	GO:0016021	integral component of membrane	141,72	138
GO:0005737	cytoplasm	12,72	10	GO:0043229	intracellular organelle	139,5	102
GO:0016020	membrane	12,23	10	GO:0043231	intracellular membrane-bounded organelle	108,22	62
GO:0043231	intracellular membrane-bounded organelle	9,96	16	GO:0044444	cytoplasmic part	101,47	52

NS- Node Score – The score is the sum of sequences directly or indirectly associated to a given GO term weighted by the distance of the term to the term of "direct annotation" i.e. the GO term the sequence is originally annotated to.
#Seqs – number of sequences annotated to the specific category

Supplementary Table 4. Primers used for qPCR analysis performed to validate results of RNA-Seq.

Gene	Symbol	Primer sequence 5'-3'	Type	Product
EF1-α	EF1-A F	CCCCTCCAGGACGTTTACAAA	Endogenous control	Bland et al[59]
	EF1-A R	CACACGGCCCACAGGTACA		
EGR1	EGR1 F	GCGGCCAGTGTAGGAGATAG	Studied	130bp
	EGR1 R	GGAGCCAGGAGAGCAGTATG		
IER2	IER2 F	AGAGAGAGGAGCTGGGAACC	Studied	107bp

Supplementary Table 5. Comparison of expression levels of *EGR1* gene detected by RNA-Seq and qPCR analysis in the non-irradiated eggs and eggs irradiated with 175 Gy and 350 Gy.

Sample	Group	qPCR expression			NGS1[B] expression			NGS2[C] expression			NGS[D] expression		
		Expression	Mean	Fold Change[E]	Transcript C27475_c0_seq1	Mean	Fold Change[E]	Transcript C27383_c0_seq1	Mean	Fold Change[E]	Sum	Mean	Fold Change[E]
0.1	Non-irradiated	3.12	3.90	-	0.00	1.06	-	0.00	0.49	-	0.00	1.54	-
0.2		7.22			0.00			0.00			0.00		
0.3		4.18			2.28			0.00			2.28		
0.4		1.06			1.94			1.94			3.88		
1.1	175Gy[A]	18.80	28.23	7.25	26.12	23.52	22.26	25.12	22.02	45.38	51.25	45.54	29.54
1.3		38.96			22.72			19.23			41.95		
1.4		26.95			21.71			21.71			43.42		
2.1	350Gy	35.98	26.88	0.95	17.84	19.20	0.82	15.14	16.24	0.74	32.97	35.44	0.78
2.2		29.43			30.73			27.24			57.97		
2.3		16.78			20.48			17.41			37.89		
2.4		25.31			7.76			5.18			12.94		
Correlation coefficient*		-	-	-	0.765	0.991	-	0.728	0.977	-	0.748	0.985	-

*-correlation coefficient was calculated for gene expression in individual samples as well as for average expression per group for both applied methods (RNA-Seq and qPCR).

[A] – 175Gy group with exclusion of one sample being an outlier and shoving high deviations in the obtained Ct values.

[B] – Gene expression for transcript 1

[C] - Gene expression for transcript 2

[D] – Sum of gene expression for both transcripts

[E] – in relation to group specified above

Supplementary Table 6. Comparison of expression levels of *IER2* gene detected by RNA-Seq and qPCR analysis in the non-irradiated eggs and eggs irradiated with 175 Gy and 350 Gy

Sample	Group	qPCR expression			NGS1[A] expression			NGS2[B] expression			NGS[C] expression		
		Expression	Mean	Fold Change[D]	Transcript C27475_c0_seq1	Mean	Fold Change[D]	Transcript C14862_c0_seq1	Mean	Fold Change[D]	suma	Mean	Fold Change[D]
0.1	Non-irradiated	1.92	3.47	-	10.63	9.56	-	8.50	8.49	-	19.13	18.05	-
0.2		5.15			11.89			10.70			22.59		
0.3		5.79			9.90			9.90			19.80		
0.4		1.00			5.82			4.85			10.68		
1.1	175Gy	8.63	5.19	1.50	45.22	76.45	8.00	42.20	72.27	8.51	87.42	148.72	8.24
1.2		3.33			139.93			134.00			273.93		
1.3		5.07			56.81			51.56			108.37		
1.4		3.72			63.85			61.29			125.14		
2.1	350Gy	3.70	4.36	0.84	49.73	68.91	0.90	47.03	65.64	0.91	96.76	134.56	0.90
2.2		3.06			176.01			166.93			342.93		
2.3		7.79			26.63			26.63			53.26		
2.4		2.89			23.29			22.00			45.29		
Correlation coefficient*		-	-	-	-0.123	0.922	-	-0.118	0.919	-	-0.120	0.920	-

*-correlation coefficient was calculated for gene expression in individual samples as well as for average expression per group for both applied methods (RNA-Seq and qPCR) (RNA-Seq and qPCR).

[A] – Gene expression for transcript 1
[B] - Gene expression for transcript 2
[C] – Sum of gene expression for both transcripts
[D] – in relation to group specified above
