# Supplementary material for: Induced androgenetic development in rainbow trout and transcriptome analysis of irradiated eggs
Source: Sci Rep. 2019 May 30;9:8084. doi: 10.1038/s41598-019-44568-7 (PMC6542805; doi:10.1038/s41598-019-44568-7)

## Supplementary File 7

## Induced androgenetic development in rainbow trout and transcriptome analysis of irradiated eggs

Konrad Ocalewicz^1^*, Artur Gurgul^2^, Klaudia Pawlina-Tyszko^2^, Tomasz Szmatoła^2, 3^, Igor Jasielczuk^2, 3^, Monika Bugno-Poniewierska^4^, Stefan Dobosz^5^,

**Supplementary File 7.** Biological processes associated with genes that were downregulated in the fertilized irradiated (350 Gy) rainbow trout eggs.


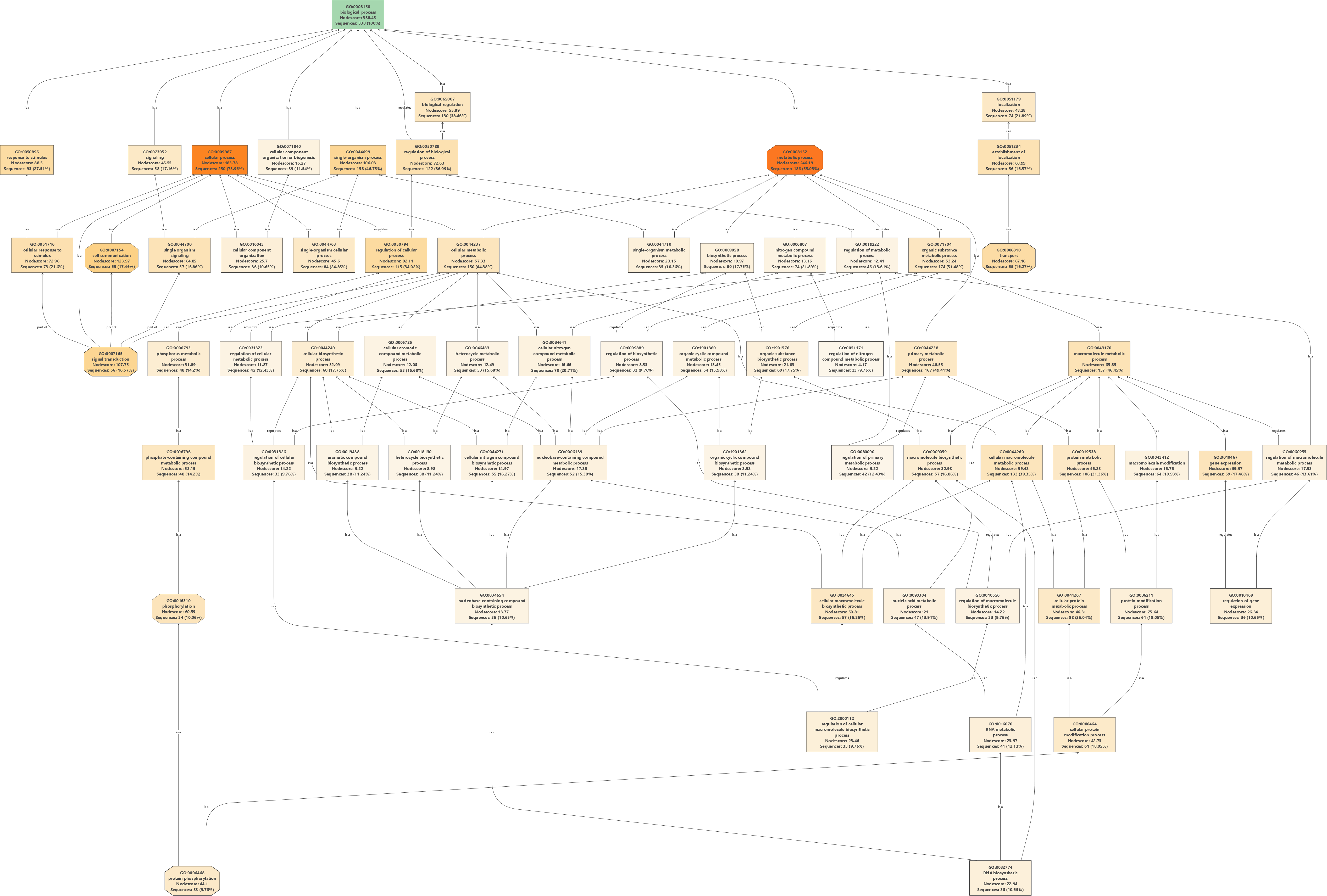

Supplement: Supplementary file 6 — Dataset 6 [file 41598_2019_44568_MOESM6_ESM.doc]
